# Effect of intrathoracic oscillations on pulmonary functions in children with cerebral palsy

**DOI:** 10.1016/j.jtumed.2023.08.003

**Published:** 2023-08-30

**Authors:** Mahmood D. Al-Mendalawi

**Affiliations:** Department of Paediatrics, Al-Kindy College of Medicine, University of Baghdad, P.O.Box 55302, Baghdad Post Office, Baghdad, Iraq

*Dear Editor*,

On using spirometry, El-Moatasem and Abbass^1^ investigated in a case–control study whether intrathoracic oscillations (IO) could influence lung function among Egyptian children with cerebral palsy (CP). Statistically significant differences were found between the cases group and control group considering various components of pulmonary function tests (PFT). Accordingly, they concluded that IO might improve lung function in children with CP.^1^ In addition to the few study limitations mentioned by El-Moatasem and Abbass,^1^ we believe that the following limitation is worthy to be considered. It is important to note that testing pulmonary function helps in the diagnosis and management of different lung conditions. Being a simple, easy-to-perform, and non-invasive tool, spirometry offers diagnostic data as reliable as testing done in a lung function laboratory. It is often used to monitor the progression of pulmonary disease and response to treatment. However, the utility of spirometry relies upon reproducibility and standardization. The predicted spirometric equations (PSE) based on various variables such as age, weight, and height are usually employed to accurately interpret the spirometric reading of different components of PFT in research and clinical setups^2^^,^^3^ and numerous pediatric population-specific PSE have been formulated.^4^, ^5^, ^6^ Regrettably, El-Moatasem and Abbass^1^ didn't explicitly specify pediatric PSE utilized in the methodology. As a result, this methodological limitation might further demolish the findings and conclusion of the study.

## Source of funding

This research did not receive any specific grant from funding agencies in the public, commercial, or not-for-profit sectors.

## Conflict of interest

The authors have no conflict of interest to declare.

## Ethical approval

Not applicable.

## Author's contributions

The author constructed the manuscript, reviewed the literature, wrote and approved the final draft, and is responsible for the content and similarity index of the manuscript.
